# Hypokalemia-Induced Abnormal Movements: Case Report

**DOI:** 10.5812/traumamon.12016

**Published:** 2013-10-13

**Authors:** Alireza Baratloo, Alaleh Rouhipour, Mohammadmahdi Forouzanfar, Farhad Rahmati, Behrooz Hashemi

**Affiliations:** 1 Department of Emergency Medicine, Shohada Tajrish Hospital, Shahid Beheshti University of Medical Sciences, Tehran, IR Iran; 2 Department of Pediatrics, Vali Asr Hospital, Ghazvin University of Medical Sciences, Abyek, IR Iran

**Keywords:** Hypokalemia, Arrhythmias, Cardiac, Seizures

## Abstract

**Introduction:**

A sudden loss of consciousness followed by abnormal movements can be ictal or syncopal in origin. Transient response by the brain to sudden decrease of blood flow may cause sudden loss of consciousness followed by abnormal movements that mimic seizure. Dysrhythmia is one of the important and critical reasons of such events that should be differentiated from seizure.

**Case Presentation:**

In this case report we describe a 55 year-old woman admitted to our emergency department first with the impression of seizure. Eventually, it was realized that she had suffered from brain hypo-perfusion secondary to hypokalemia induced arrhythmia. Her arrhythmia was managed by unsynchronized biphasic shock in acute phase and also potassium replacement. She was then admitted to the CCU (Coronary Care Unit) where she received further care for medical management and drug dose adjustment and was discharged 4 days later.

**Conclusions:**

Syncope from arrhythmia most commonly results from ventricular tachycardia, which accounts for 11% of all cases of syncope. Torsades de point is a unique type of ventricular tachycardia, characterized by QRS complexes of changing amplitude proceeded by prolonged QT intervals and almost often followed by loss of consciousness and also seizure like movements. Prolonged QT interval which is an important provocative factor for torsades de point commonly results from interactions between drug therapy, myocardial ischemia, and electrolyte disturbances such as hypokalemia or hypomagnesaemia. Changes in the extracellular potassium level have predominant and profound influences on the function of the cardiovascular system that may provoke fatal demonstrations such as QT prolongation, ventricular arrhythmia and even cardiac arrest. Electrolyte assessment is particularly important in certain patient populations, such as the elderly in whom a variety of pathological states or conditions like dehydration or renal failure are more common. Early identification and correction of these disturbances are necessary to control either seizures or seizure-like movements and prevent permanent brain damage, as anticonvulsants alone are generally ineffective.

## 1. Introduction

Approach to seizing patient in the Emergency Department (ED), pulse checking should be one of the first priorities to differentiate abnormal movement resulting from cerebral hypoxia or hypo-perfusion from an actual seizure. Missing the actual etiology of abnormal movements may lead to inappropriate use of antiepileptic drugs, in addition to useless and expensive paraclinical tests (1, 2).Transient response by the brain to sudden decrease of blood flow may cause sudden loss of consciousness followed by abnormal movements that mimic seizure. Dysrhythmia is one of the most important and critical reasons of such events that should be differentiated from seizure (2, 3). Rhythm disturbances are among the most frequent and potentially hazardous causes of syncope and dizziness. Syncope from arrhythmia may result from ventricular tachycardia, which accounts for 11% of all cases. Polymorphic ventricular tachycardia related to long QT intervals which is also called “Torsades de point” is a well-recognized cause of syncope and sudden death (4).

## 2. Case Presentation

**Figure 1.**
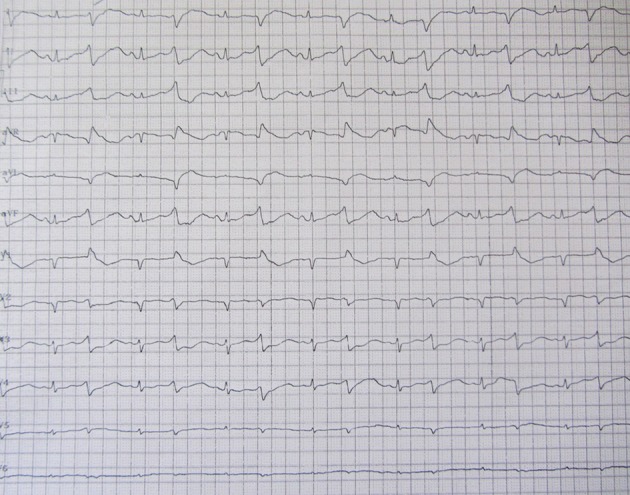
Obvious QT Prolongation. 50/min heart rate, normal axis, no significant ST-T change, 0.76 msec QT corrected time

**Figure 2.**
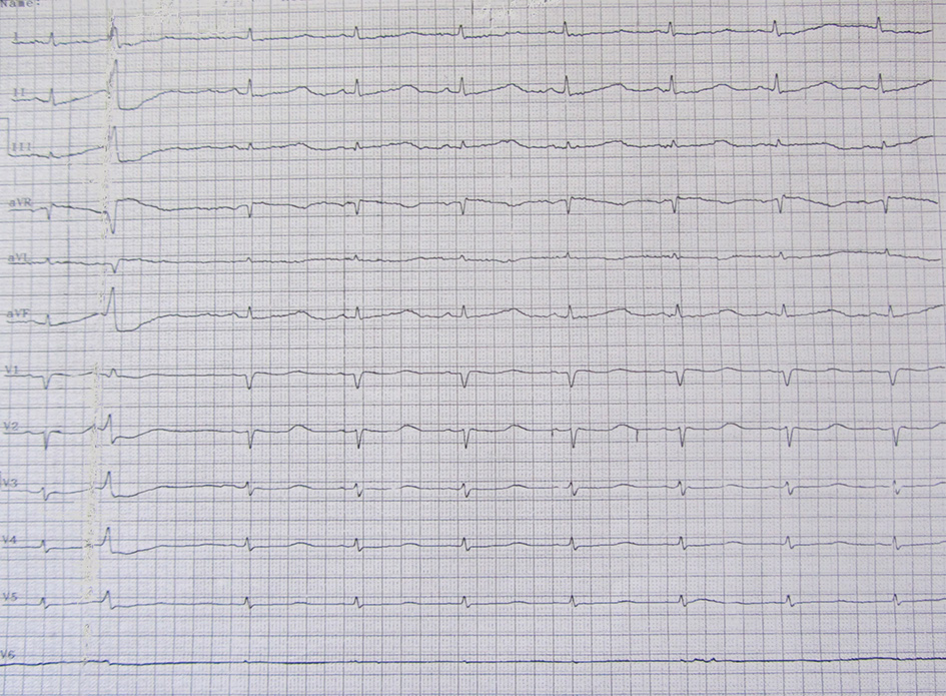
Obvious QT Prolongation and Bigeminy PVC. 50/min heart rate, normal axis, no significant ST-T change, 0.76msec QT corrected time, bigeminy PVC

A 55 year-old woman was admitted to our emergency department due to her first episode of seizure. A generalized tonic colonic seizure occurred 30 min. before her arrival and lasted for 3 minutes followed by a 20 minute postictal period. On admission to ED, she was alert with complaints of chest discomfort and dyspnea. She had a history of breast cancer for which had undergone a surgery 5 months ago followed by a 2 month course of chemotherapy. She also was a known case of hypertension and hypothyroidism. Levothyroxine, levothyronine, furosemide, hydrochlorothiazide, sertraline, clonazepam, buspirone, triamterene-H and dimenhydrinate were the drugs that she was on but not taking regularly. On admission to ED she had axillary temperature of 36.5C, 14/min respiratory rate, 62/min pulse rate and 110/70 mmHg blood pressure, O2sat=96% in room air and blood sugar glucometer was 110mg/dl. On laboratory examination, Na=129mEq/L, K=2.5 mEq/L, Ca=10 mg/dl, P=3.1 mg/dl and Mg=2.6 mEq/L were reported and also in ABG, PH=7.77, PCO2=27 mmHg and HCO3=39.7mEq/L were seen. Emergency computed tomography of the brain was normal. In ECG she had obvious QT prolongation (Figure 1) which progressed to bigeminy PVC (Figure 2). 

In the ED, she had a seizure again accompanied with polymorphic ventricular tachycardia (VT) that was cardioverted via 100 joules unsynchronized biphasic shock. Normal saline (0.9%) infusion was started to correct hyponatremia. Intravenous potassium replacement was initiated to correct hypokalemia and reverse the intracellular shift of hydrogen ions and reduce cellular acidosis as well as enhance HCO3excretion in the urine. Spironolactone was then administrated due to diagnosis of saline-resistant metabolic alkalosis to reduce mineralocorticoid activity. She was admitted to CCU for the adjustment of her drugs and was discharged 4 days later.

## 3. Conclusions

Torsades de point is a unique type of ventricular tachycardia, characterized by QRS complexes of changing amplitude, proceeded by a prolonged (> 500 msec) QT interval, most frequently observed with anti-arrhythmic drug therapy and electrolyte disturbances; almost often followed by loss of consciousness and seizure-like movements (5). Torsades de point literally meaning the “twisting of points”, refer to the repeating continuum of upward then downward pointing ventricular complexes. As the morphology of the QRS complex can vary with each beat, the outline on an ECG looks like a twisted ribbon (6). The underlying basis for rhythm disturbance is delay in phase III of the action potential, mediated by the potassium channels. This delay in depolarization allows for the emergence of re-entry points, called the “R-on-T” phenomenon. This vulnerable period in ventricular repolarization is a time when some, but not all, of the myocardial tissue is ready for the signal (7). Prolonged QT intervals an important provocative factor for Torsades de point, can either be inherited (tachycardia dependent) as congenital mutations of ion channels carrying the cardiac impulse/action potential or acquired (pause dependent) as a result of drugs which block these cardiac ion currents, with the latter being more common. Acquired QT prolongation and torsades are often multifactorial, including interactions between drug therapy, myocardial ischemia, and electrolyte disturbances such as hypokalemia (5). Electrolyte abnormalities may affect many organs and tissues, including the brain. Most of the clinical manifestations of these derangements are predominantly neurologic and parallel the severity of neuronal damage. Furthermore, these disorders may appear with seizures, or with rapidly progressive neurologic symptoms and signs, and thus require emergency treatment (8, 9). Acute and/or severe electrolyte imbalances frequently cause seizures, and these seizures may be the sole presenting symptom (Table 1). Seizures are especially common in patients with sodium disorders, hypocalcemia, and hypomagnesemia (8, 10). Unlike other electrolyte alterations, hypokalemia or hyperkalemia rarely causes symptoms in the CNS, and seizures do not occur (8). The majority of patients with mild hypokalemia (3.0 - 3.5 mmol/L) are asymptomatic and initial symptoms, when they occur, may be non-specific such as weakness or fatigue (11). Signs and symptoms become more apparent as the potassium level drops below 3.0mmol/L, however, in patients where the level has decreased rapidly, or the patient is at risk of arrhythmia, even a mild decrease in potassium may result in significant clinical problems (12). 

**Table 1.** Electrolyte Abnormality and Seizures in Clinical Practice

Electrolyte Abnormality	Frequency in Clinical Practice	Frequency of Seizures in Acute/Severe Imbalance
**Hyponatremia**	+++	++
**Hypernatremia**	++	++/+
**Hypocalcemia**	+	++/+
**Hypercalcemia**	++	+
**Hypomagnesemia**	++	++/+
**Hypokalemia**	+++	−
**Hyperkalemia**	++	−

Some studies showed that reduction in potassium level increases the probability of ventricular tachycardia after acute myocardial infarction. However, in most of these studies, patients were divided into two groups with normal potassium or below normal level and relative importance of different potassium levels in prevalence of arrhythmias as well as the involvement of other confounding factors such as consumption of drugs were not evaluated. The study of Pourmoghaddas et al. showed that the risk of ventricular tachycardia after acute myocardial infarction in the group with potassium level less than 3.8 mEq/L was almost two folds higher than the group with potassium level between 3.8 to 4.5 mEq/L; however the risk of ventricular tachycardia between the two groups of potassium level greater than 4.5mEq/L and the group with potassium level between 3.8 to 4.5 mEq/L had no significant difference. The relative risk of ventricular tachycardia occurrence in the group with potassium level more than 4.5mEq/L was 1.03 folds than the group with potassium level between 3.8 and 4.5 mEq/L. Therefore, maintaining the serum potassium at the range of more than 3.8mEq/l would minimize the risk of ventricular tachycardia (13). Changes in the extracellular potassium level have predominant and profound influences on the function of the cardiovascular system that may be accompanied with hypotension, bradycardia or tachycardia, premature atrial or ventricular beats and also may provoke fatal manifestations, such as QT prolongation, ventricular arrhythmias and even cardiac arrest (14). While hypokalemia has been commonly associated with torsades de point, it is usually chronic hypokalemia with concomitant antiarrhythmic drug administration and/or hypomagnesaemia. So electrolyte screening is particularly important in certain patient populations, such as the elderly whom a variety of pathological states or conditions like dehydration or renal failure are more common (15). The majority of adult cases of torsades is acquired and pause dependent (Table 2). These episodes of torsades are precipitated by a slow heart rate. While many episodes of torsades terminate spontaneously, some degenerate to a more typical ventricular fibrillation resulting in death (16). Treatment of intermittent torsades in stable patients is based on correcting any underlying metabolic or electrolyte abnormalities (5). 

**Table 2.** Causes of Acquired and Pause Dependent QT Prolongation Resulting in Torsades

**Drug induced:** Class lA and lC antidysrhythmics; many phenothiazines/butyrophenones (notably haloperidol and droperidol), cyclic antidepressant, antibiotics (especially macrolides), organophosphate, antihistamines, antifungals, antiseizure and antiemetic agents
**Electrolyte abnormalities:**Hypokalemia, hypomagnesemia, hypocalcemia (rarely)
**Diet related:**Starvation, low protein
**Severe bradycardia or atrioventricular block**
**Hypothyroidism**
**Contrast injection**
**Cerebrovascular accident, especially intraparenchymal**
**Myocardial ischemia**

Magnesium replacement acts by shortening the QT interval and suppressing early depolarization. In chronic hypokalemia a 42% incidence of hypomagnesemia has been reported. Ventricular arrhythmias are commonly associated with combined hypokalemia and hypomagnesemia. Under these conditions, magnesium and potassium replacement are required for ventricular arrhythmia control. While torsades has been commonly reported with normal serum magnesium concentrations, exogenous magnesium administration has been shown to be effective in terminating torsades despite normal serum levels. Some studies found no correlation between muscle and serum magnesium concentrations in chronically ill patients, thus a normal serum magnesium concentration may be found with total body hypomagnesemia (16).Because neurologic symptoms of electrolyte disorders are generally functional rather than structural, the neurologic manifestations of electrolyte disturbances are typically reversible (15). However, because functional dysfunctions such as seizures can lead to structural alterations, it is important to treat the underlying disturbance before the pathology becomes permanent (8). Seizure often represents an important clinical electrolyte disturbance, especially in sodium, calcium and magnesium but unlike other electrolyte alterations, hypokalemia or hyperkalemia rarely cause symptoms in the CNS; on the other hand, potassium abnormality may provoke fatal arrhythmias. If the patient is seizing in the ED, the first step is to confirm that a pulse is present and that the seizure like activity is not the result of cerebral hypoxia from lack of blood flow. A sudden loss of consciousness followed by abnormal movements can be ictal or syncopal in origin, or may be due to dysrhythmia such as torsades de point. Hypokalemia and hypothyroidism are two of the main acquired causes of prolonged QT syndromes that can produce torsades. Early identification and correction of these disturbances are necessary to control seizures and prevent permanent brain damage, though anticonvulsants alone are generally ineffective. All physicians should be aware of these clinical conditions and consider underlying medical disorders to initiate rapid appropriate therapy.
